# Evidence for Chronic Low-Grade Systemic Inflammation in Individuals with Agoraphobia from a Population-Based Prospective Study

**DOI:** 10.1371/journal.pone.0123757

**Published:** 2015-04-13

**Authors:** En-Young N. Wagner, Jan T. Wagner, Jennifer Glaus, Caroline L. Vandeleur, Enrique Castelao, Marie-Pierre F. Strippoli, Peter Vollenweider, Martin Preisig, Roland von Känel

**Affiliations:** 1 Division of Psychosomatic Medicine, Inselspital, Bern University Hospital, Bern, Switzerland; 2 Department of Neurology, Inselspital, Bern University Hospital, Bern, Switzerland; 3 Swissmedic, Division Clinical Review, Bern, Switzerland; 4 Department of Psychiatry, Center for Research in Psychiatric Epidemiology and Psychopathology, Lausanne University Hospital, Prilly, Switzerland; 5 Department of Medicine, Internal Medicine, Lausanne University Hospital, Lausanne, Switzerland; 6 Department of Psychosomatic Medicine, Clinic Barmelweid, Barmelweid, Switzerland; University of Illinois-Urbana Champaign, UNITED STATES

## Abstract

**Background:**

Anxiety disorders have been linked to an increased risk of incident coronary heart disease in which inflammation plays a key pathogenic role. To date, no studies have looked at the association between proinflammatory markers and agoraphobia.

**Methods:**

In a random Swiss population sample of 2890 persons (35-67 years, 53% women), we diagnosed a total of 124 individuals (4.3%) with agoraphobia using a validated semi-structured psychiatric interview. We also assessed socioeconomic status, traditional cardiovascular risk factors (i.e., body mass index, hypertension, blood glucose levels, total cholesterol/high-density lipoprotein-cholesterol ratio), and health behaviors (i.e., smoking, alcohol consumption, and physical activity), and other major psychiatric diseases (other anxiety disorders, major depressive disorder, drug dependence) which were treated as covariates in linear regression models. Circulating levels of inflammatory markers, statistically controlled for the baseline demographic and health-related measures, were determined at a mean follow-up of 5.5 ± 0.4 years (range 4.7 – 8.5).

**Results:**

Individuals with agoraphobia had significantly higher follow-up levels of C-reactive protein (p = 0.007) and tumor-necrosis-factor-α (p = 0.042) as well as lower levels of the cardioprotective marker adiponectin (p = 0.032) than their non-agoraphobic counterparts. Follow-up levels of interleukin (IL)-1β and IL-6 did not significantly differ between the two groups.

**Conclusions:**

Our results suggest an increase in chronic low-grade inflammation in agoraphobia over time. Such a mechanism might link agoraphobia with an increased risk of atherosclerosis and coronary heart disease, and needs to be tested in longitudinal studies.

## Introduction

Increasing evidence suggests that anxiety disorders are linked to elevated levels of circulating biomarkers indicating low-grade inflammation. Based on experimental data, it has been hypothesized that it is particularly the experience of acute stress in anxiety states, which leads to these increments [1]. Most of the research on the association between anxiety disorders and inflammation stems from studies on posttraumatic stress disorder (PTSD) and the referring potential pathophysiological pathways have extensively been described [2–5]. It is assumed, that a prolonged or excessive activation of the acute stress response system in PTSD may increase the risk for excessive systemic inflammation [6, 7] by long-term adaptations of bodily systems. One such adaptation might consist of a decrease of cortisol levels and a consecutively insufficient glucocorticoid signaling, causing excessive levels of cell-mediated and proinflammatory cytokines, as well as excessive stimulation of the hypothalamic-pituitary-adrenal (HPA) axis [8–10].

However, it may also well be that the presence of a proinflammatory state precedes the actual development of PTSD [7, 11]. Since not all trauma-exposed individuals subsequently develop PTSD [12], it is hypothesized that the development of PTSD is associated with biological vulnerability factors, which are already present before the onset of symptoms. In a recent review, such pre-existing vulnerability factors in the glucocorticoid signaling pathway for the development of PTSD have been presented [5].

Sparse evidence from relatively small clinical studies also suggests that inflammatory activity is increased in patients with panic disorder [13] and generalized anxiety disorder [14].

In comparison to PTSD, knowledge about the association between inflammation and agoraphobia is scarce—despite the fact that agoraphobia is one of the most prevalent, persistent and disabling of the mental disorders [15], with a well-known poor prognosis and frequent incomplete remission [15, 16]. Therefore it is a lifetime diagnosis in most of the cases. The main clinical feature of agoraphobia is the presence of anxiety about being in places or situations from which escape may be difficult (or embarrassing) or in which help may not be available in the case of a panic attack or the occurrence of panic-like symptoms [17, 18].

Two studies that have investigated inflammation in patients with agoraphobia and panic attacks so far did not show increased levels of inflammatory markers. However, measurements in one small study on 36 patients with agoraphobia and panic attacks were performed after in vitro stimulation of immune competent cells and, moreover, might be confounded by a four-arm pharmacological intervention design [19]. The other recent population-based study found no significant associations of circulating levels of C-reactive protein (CRP), tumor necrosis factor (TNF)-α, and interleukin (IL)-6 in individuals with a current panic disorder and/or agoraphobia [20]. However, that study did not investigate an association between inflammation and agoraphobia separately and applied a cross-sectional design [20].

To date, there is no study that has investigated whether patients with agoraphobia show increased low-grade inflammation compared to individuals without agoraphobia, and whether agoraphobic individuals show an increase of low-grade inflammation over time in comparison to their non-agoraphobic counterparts, reflecting a cumulative and progressive immune function dysregulation.

The following study aimed to address this existing research gap by examining the association between agoraphobia and low-grade inflammation in a large population based sample. Thereby, changes in circulating levels of inflammatory markers, that is CRP, IL-1β, IL-6, TNF-α, and adiponectin, in patients with agoraphobia compared to those without this anxiety disorder were assessed prospectively, taking potential confounding factors commonly associated with inflammation activity, and other relevant psychiatric disorders (including other anxiety disorders), into account. We hypothesized that individuals with a lifetime diagnosis of agoraphobia might have higher levels of proinflammatory markers and lower levels of the “cardioprotective” adipo(cyto)kine adiponectin [21] than non-agoraphobic individuals and that these differences would increase over time.

## Materials and Methods

### Ethics statement

The Institutional Ethics Committee of the University of Lausanne approved the CoLaus and subsequently the PsyCoLaus study. All participants signed a written informed consent after having received a detailed description of the goal and funding of the study.

### Study participants and design

The data of the present paper stemmed from CoLaus|PsyCoLaus [22, 23], a cohort study designed to prospectively assess mental disorders and cardiovascular risk factors (CVRFs) in the general population. Blood and plasma samples were also collected for the study of biomarkers and genetic variants.

The study participants were selected from a random sample of 19’830 residents (35%) of the city of Lausanne, in the age range of 35 to 75 years, derived from the electronic database of the entire population of the 1st January 2003. Letters were sent to all subjects and among respondents (n = 15’109) a final sample of 6736 subjects agreed to participate in CoLaus after having received additional information regarding the study. The baseline somatic assessment for the CoLaus study (n = 6736) was conducted between June 2003 and May 2006 and has been described in detail elsewhere [22]. In a second step, we asked the 5535 subjects aged between 35 and 66 years to participate in PsyCoLaus. Subsequently, 67% of them accepted to take part in the psychiatric evaluation, and the final sample was comprised of 3719 individuals who underwent both the somatic and psychiatric exams (PsyCoLaus study) [23]. Ninety-two percent of them were Caucasians. The gender distribution of the PsyCoLaus sample (47% men) did not significantly differ from that of the general population in the same age range (mean age ± SD: 50.9 ± 8.8 years). Although the youngest 5-year band of the cohort was underrepresented and the oldest 5-year band overrepresented, participants of PsyCoLaus (mean age ± SD: 50.9 ± 8.8 years) and individuals who refused to participate revealed comparable scores on the General Health Questionnaire [24], French translation [25], a self-rating instrument completed at the somatic exam.

Among the 3174 participants who had complete data for agoraphobia from the psychiatric evaluation at baseline, the majority also took part in the somatic follow-up evaluation. At follow-up 3100, 2792, 2792, 2792, and 2679 subjects provided a blood sample for measurements of the pro-inflammatory markers CRP, IL-1β, IL-6, TNF-α, and adiponectin, respectively. Participants with high-sensitivity CRP > 10 mg/L (n = 210) at baseline and follow-up were excluded from the analysis, as these values are likely to be a sign of acute infection, resulting in a final study sample of 2890 subjects. The average duration between the somatic baseline and follow-up assessments was 5.5 ± 0.4 years (range 4.7–8.5). The average duration between somatic and psychiatric evaluations at baseline was 1.3 ± 0.5 years (0.2–5.7). There was no significant difference in time from somatic to psychiatric evaluation (p = 0.326) and time to the follow-up assessment (p = 0.414) for agoraphobics compared to the rest of the sample (Table 1).

**Table 1:** Baseline characteristics of the whole sample and per group with and without agoraphobia.

	All	No agoraphobia	Agoraphobia	p value
	(n = 2890)	(n = 2766, 95.7%)	(n = 124, 4.3%)	
**Duration between somatic and psychiatric evaluations at baseline, years**	1.3±0.5 (0.2–5.7)	1.3±0.5 (0.2–5.7)	1.3±0.4 (0.6–4.3)	0.326
**Length of follow-up** ^**a**^ **, years**	5.5±0.4 (4.7–8.5)	5.5±0.4 (4.7–8.5)	5.5±0.4 (5.1–8.2)	0.414
**Age, years**	49.7±8.8 (35.0–66.6)	49.6±8.8 (35.0–66.6)	50.6±8.4 (35.7–65.9)	0.227
**Socioeconomic status** ^**b**^	3.4±1.3 (1.0–5.0)	3.5±1.3 (1.0–5.0)	3.2±1.3 (1.0–5.0)	0.040*
**Hypertension** ^**c**^				0.990
No	2309 (79.8%)	2207 (79.8%)	99 (79.8%)	
Yes	584 (20.2%)	559 (20.2%)	25 (20.2%)	
**Body mass index**				0.205
Underweight (BMI<18.5 kg/m^2^)	49 (1.7%)	44 (1.6%)	5 (4.0%)	
Normal (18.5 kg/m^2^≤BMI<25 kg/m^2^)	1504 (52.0%)	1440 (52.1%)	64 (51.6%)	
Overweight (25 kg/m^2^≤BMI<30 kg/m^2^)	1002 (34.7%)	959 (34.7%)	43 (34.7%)	
Obesity (BMI≥30 kg/m^2^)	335 (11.6%)	323 (11.7%)	12 (9.7%)	
**Glucose, fasting state mmol/L**	5.5±1.0 (0.3–21.2)	5.5±1.0 (0.3–21.2)	5.5±1.1 (4.2–13.8)	0.598
**Total cholesterol/HDL-cholesterol ratio**	3.6±1.1 (1.4–12.0)	3.6±1.1 (1.4–12.0)	3.5±1.1 (1.8–7.7)	0.574
**Smoking status**				0.040*
Never	1140 (39.5%)	1102 (39.8%)	38 (30.7%)	
Former/Current	1750 (60.5%)	1664 (60.2%)	86 (69.3%)	
**Alcohol consumption**				0.032*
Nondrinkers	713 (24.7%)	674 (24.4%)	39 (31.5%)	
Low (1–6 drinks/week)	1118 (38.7%)	1068 (38.6%)	50 (40.3%)	
Moderate (7–13 drinks /week)	594 (20.6%)	568 (20.5%)	26 (21.0%)	
High (14+ drinks /week)	465 (16.1%)	456 (16.5%)	9 (7.3%)	
**Leisure physical activity** ^**d**^				0.265
No	1281 (44.3%)	1220 (44.1%)	61 (49.2%)	
Yes	1609 (55.7%)	1546 (55.9%)	63 (50.8%)	
**Major depressive disorder**				<0.001**
No	1630 (56.4%)	1581 (57.2%)	49 (39.5%)	
Yes	1260 (43.6%)	1185 (42.8%)	75 (60.5%)	
**Other anxiety disorders** ^**e**^				<0.001**
No	2297 (79.5%)	2239 (80.9%)	58 (46.8%)	
Yes	593 (20.5%)	527 (19.1%)	66 (53.2%)	
**Drug dependence** ^**f**^				0.334
No	2812 (97.3%)	2693 (97.4%)	119 (96.0%)	
Yes	77 (2.3%)	72 (2.6%)	5 (4.0%)	

Data are given as mean ± standard deviation (range) or percentage value.

Statistical analyses used Mann-Whitney-U or Pearson’s chi-square test (* p < 0.05, ** p < 0.001).

SES, socioeconomic status; HDL, high-density lipoprotein; BMI, Body mass index.

^a^ Duration between somatic evaluation at baseline and somatic evaluation at follow-up.

^b^ A value of “3” represents an SES of III (middle class) on the Hollingshead Scale.

^c^ Systolic blood pressure ≥ 140 mmHg and/or diastolic blood pressure ≥ 90 mmHg.

^d^ Physically active at least or more than 20 minutes twice a week.

^e^ Generalized anxiety disorder, panic disorder, posttraumatic stress disorder, and/or social phobia.

^f^ Marijuana, cocaine, solvent, hallucinogen, stimulant, sedative or/and narcotic dependence.

### Assessment of agoraphobia

Diagnostic information on lifetime agoraphobia was collected using the semi-structured Diagnostic Interview for Genetic Studies (DIGS) [26]. The DIGS also assessed the severity of agoraphobia by a Global Assessment of Functioning score (GAF score) specific to the agoraphobia section. The DIGS was developed by the National Institute of Mental Health (NIMH) Molecular Genetics Initiative to obtain a more precise assessment of phenotypes through a wide spectrum of DSM-IV Axis I criteria. Psychiatric diagnoses were assigned according to the DSM-IV. We used the French translation of the DIGS [27] with excellent inter-rater reliability in terms of kappa and Yule’s Y coefficients for major mood and psychotic disorders [28] as well as for substance use disorders [29], and 6-week test-retest reliability which was somewhat lower but still in the fair to good ranges [28, 29]. The inter-rater reliability for specific anxiety disorders was very good, whereas the 6-week test-retest reliability estimates were in the fair or good ranges [30]. Interviewers were required to be psychologists or psychiatrists, who were trained over a 2-month period. Their training included rating tapes and supervised co-ratings. To provide ongoing supervision throughout the study, each interview and diagnostic assignment was reviewed by an experienced senior psychologist. The psychiatric investigation was conducted approximately one year after the baseline somatic examination.

### Assessment of proinflammatory markers

Blood sampling to determine inflammatory levels took place at both the baseline and follow-up assessments. Venous blood samples (50 mL) were drawn in the fasting state and allowed to clot. Serum was preferred to plasma, as it has been shown that different anticoagulants may affect absolute cytokine levels differently [31, 32]. High-sensitivity CRP was assessed by immunoassay and latex HS (IMMULITE 1000-High, Diagnostic Products Corporation, LA, CA, USA) with maximum intra- and interbatch coefficients of variation of 1.3% and 4.6%, respectively. Serum samples were kept at -80°C before assessment of IL-1β, IL-6, and TNF-α and sent on dry ice to the laboratory. Levels of these cytokines were measured using a multiplexed particle-based flow cytometric cytokine assay [33].

This methodology yields cytokine concentrations correlating well with those obtained by other methods such as ELISA [34, 35]. Milliplex kits were purchased from Millipore (Zug, Switzerland). The procedures closely followed the manufacturer’s instructions. The analysis was conducted using a conventional flow cytometer (FC500 MPL, BeckmanCoulter, Nyon, Switzerland). Good agreement between signal and cytokine was found within the assay range (R^2^ ≥ 0.99). Intra- and inter-assay coefficients of variation (CV) were respectively 15% and 16.7% for IL-1β, 16.9% and 16.1% for IL-6 and 12.5% and 13.5% for TNF-α. Adiponectin was assessed by ELISA (R&D Systems, Inc, Minneapolis, USA) with a maximum inter-assay CV of 8.3% and a maximum intra-assay CV of 8.3%. For quality control, repeated measurements were conducted in 80 subjects randomly drawn from the initial sample.

### Assessment of covariates

The somatic exam comprised measurements of body weight, height and blood pressure (triplicate measure on the left arm after at least a 10-min rest in the seated position). Venous blood samples were drawn from each participant after an overnight fast to measure the levels of glucose, total cholesterol, and high-density lipoprotein (HDL)-cholesterol. Continuous values of fasting blood glucose and the total cholesterol/HDL-cholesterol-ratio were used. According to the World Health Organization guidelines, overweight was defined as a body mass index (BMI) of 25 and to 30 kg/m^2^ and obesity was defined as a BMI ≥ 30 kg/m^2^ [36]. A diagnosis of hypertension was assigned in the case of systolic blood pressure ≥ 140 mmHg and/or diastolic blood pressure ≥ 90 mmHg. Information on gender and age, socioeconomic status (SES), physical inactivity and nicotine and alcohol consumption was taken from the DIGS. SES was assessed using the Hollingshead scale [37]. A subject was considered as physically active when he or she reported regular physical exercise for at least 20 minutes twice a week. Regular nicotine consumption was defined as a current or past history of smoking. Alcohol intake was assessed by self-reported alcohol consumption in the last seven days, expressed as the number of standard drinks. A standard drink was defined as a glass of wine, a bottle of beer or a shot of spirits, approximating 10–12 g ethanol [38]. Subjects were categorized as nondrinkers, low (1–6 drinks/week), moderate (7-13/week) and high (14+/week) alcohol consumers. Questionnaire-based data on alcohol consumption have been shown to correlate well with daily alcohol intake over the previous 4 years [39]. Lifetime major depressive disorder (MDD), other anxiety disorders (generalized anxiety disorder, panic disorder, posttraumatic stress disorder, social phobia) and drug dependence (marijuana, cocaine, solvent, hallucinogen, stimulant, sedative or/and narcotic dependence) were collected using the DIGS.

### Statistical analysis

All statistical analyses were performed using the IBM^®^ PASW^®^ 18.0 statistical software package (IBM Corporation, New York, USA) and the Statistical Analysis System, version 9.2 for Windows (SAS Institute Inc., Cary, NC, USA). Data are presented as means ± standard deviation (range) or absolute numbers and percentages for continuous and categorical variables, respectively. We categorized the participants into two groups, those with and those without agoraphobia. To detect significant differences in covariates between the two groups, we applied the Mann-Whitney-U and Pearson’s chi-square test for continuous and categorical variables, respectively. Lower limits of detection (LOD) for IL-1β, IL-6 and TNF-α were 0.2 pg/ml. Undetectable measures for IL-1β, IL-6 and TNF-α at baseline and follow-up were replaced by half the LOD (i.e., 0.1 pg/ml) as was previously suggested [33–35]. For adiponectin, and high-sensitivity CRP, all values were detectable at baseline and at follow-up. Given that as many as 37% and 25% of the values of IL-1β at baseline and at follow-up respectively were below the LOD, we dichotomized this variable at the median and applied logistic regression models. For subsequent analyses, inflammatory measures (CRP, IL-6, TNF-α and adiponectin) were log10-transformed to normalize distributions. The sum of log10-transformed and z-scored high-sensitivity CRP, IL-6 and TNF-α were used to create a composite score of inflammatory markers at baseline and at follow-up [40]. Only participants who had data available for all three of these markers were included in these analyses (n = 2569).

Associations between agoraphobia at baseline and CRP, IL-6, TNF-α, adiponectin and the composite score of inflammatory markers outcome levels at baseline or at follow-up were determined using multiple linear regression models, separately for each outcome variable. For the associations between agoraphobia at baseline and inflammatory markers at follow-up, five models of increasing complexity were computed; first (model 1) only with an adjustment for value of the corresponding inflammatory measure at baseline and the duration between somatic evaluation at baseline and at follow-up (i.e., length of follow-up), and, subsequently (models 2–5), with the same adjustments as for model 1 and additional adjustments for demographic and health-related covariates. In model 2, we made adjustments for inflammatory measures at baseline and sociodemographic characteristics (age, gender, SES). In model 3, we additionally adjusted for physical CVRFs (hypertension, BMI, glucose, total cholesterol/HDL-cholesterol), in model 4, for behavioral CVRFs (smoking, alcohol consumption, physical activity), and finally, in model 5, for other relevant lifetime psychiatric disorders: major depressive disorders, other anxiety disorders (generalized anxiety disorder, panic disorder, posttraumatic stress disorder, social phobia) and drug dependence (marijuana, cocaine, solvent, hallucinogen, stimulant, sedative or/and narcotic dependence). Statistical significance was considered at p < 0.05. We did not adjust p-values for multiple comparisons for the following two reasons [41, 42]: a) the hypothesized relationships between agoraphobia and inflammatory measures were specified a priori; and b) the examined inflammatory measures are actual observations in nature and all indicative of the same biological process (i.e., chronic low-grade systemic inflammation).

## Results

### Characteristics of study participants

Baseline characteristics of the study sample (n = 2890) are presented in Table 1. On average, participants were 50 years old and 53% were female. Concerning CVRFs, quite a sizeable percentage of participants were former or current smokers (61%). Thirty-seven percent of participants reported moderate-to-high alcohol consumption and 44% were physically inactive. Lifetime agoraphobia was detected in 124 individuals (4.3% of all participants).

The comparison between agoraphobics and non-agoraphobics on demographic and health characteristics revealed significantly more women and more smokers, but fewer alcohol consumers of alcohol in the group with agoraphobia. Individuals with agoraphobia also showed a significantly higher prevalence of concomitant anxiety and major depressive disorders than their non-agoraphobic counterparts. The proinflammatory biomarkers at baseline (Table 2) did not differ significantly between subjects with agoraphobia and controls, even after adjustment for all covariates.

**Table 2:** Associations between lifetime agoraphobia status at baseline and inflammatory measures at baseline and follow-up.

	Inflammatory markers at baseline			Inflammatory markers at follow-up		
	No agoraphobia	Agoraphobia	p value	No agoraphobia	Agoraphobia	p value
CRP^a^ (mg/l)	0.01±0.43 (n = 2766)	0.01±0.41 (n = 124)	0.851	0.06±0.40 (n = 2766)	0.14±0.43 (n = 124)	**0.026**
Interleukin-1β^b^ (pg/ml)	47.8% (n = 2671)	46.3% (n = 121)	0.748	54.8% (n = 2671)	50.4% (n = 121)	0.343
Interleukin-6^a^ (pg/ml)	0.15±0.67 (n = 2671)	0.16±0.68 (n = 121)	0.929	0.47±0.77 (n = 2671)	0.56±0.78 (n = 121)	0.217
TNF-α^a^ (pg/ml)	0.46±0.40 (n = 2671)	0.45±0.43 (n = 121)	0.834	0.65±0.47 (n = 2671)	0.74±0.43 (n = 121)	**0.042**
Adiponectin^a^ (mg/l)	0.89±0.30 (n = 2559)	0.91±0.28 (n = 120)	0.610	0.58±0.30 (n = 2559)	0.56±0.34 (n = 120)	0.533
Composite score^a^ ^,^ ^c^	-0.07±2.02 (n = 2457)	-0.08±1.98 (n = 112)	0.963	-0.07±1.98 (n = 2457)	0.50±1.95 (n = 112)	**0.003**

Values for inflammatory measures are given as unadjusted log10-transformed means ± standard deviation, values for interleukin-1β are given as prevalence.

CRP, C-reactive protein; TNF, tumor necrosis factor.

^a^ Multiple regression with log10 transformed cytokine or CRP or adiponectin.

^b^ Logistic regression with interleukin-1β concentration dichotomized at the median.

^c^ Composite score of inflammatory markers includes CRP, interleukin-6 and TNF-α. Only participants who had data available for all three of these markers were included in these analyses.

### Inflammatory measures at follow-up

The comparison of circulating levels of inflammatory measures at baseline and follow-up between agoraphobics and non-agoraphobics is presented in Table 2 (log10-transformed means ± standard deviations, values for IL-1β are given as prevalence). Mean values of the log10-transformed biomarkers levels did not significantly differ between agoraphobics and non-agoraphobics at baseline. After adjusting for CRP at baseline and all further covariates, mean log10-transformed CRP at follow-up was higher in agoraphobics than in non-agoraphobics at baseline. This absolute increase was statistically significant (Table 3 and Table 4; β = 0.087 (95%-CI 0.024 to 0.151; p = 0.007). The effect size of agoraphobia was similar to that of overweight, but lower than those of underweight and obesity. In contrast, it was higher than the effect sizes of smoking and alcohol consumption. Similarly, TNF-α levels were significantly higher among subjects with agoraphobia than among those without and the significance of this association was preserved in all five models (Table 3 and Table 7; β = 0.089 (95%-CI 0.003 to 0.174; p = 0.042). In addition, the effect size of agoraphobia for TNF- α was higher than those of all the other covariates, except for drug dependence. In contrast to the other proinflammatory markers, the cardio-protective biomarker adiponectin was significantly lower in persons with than those without agoraphobia after adjustment for covariates (models 1–5; Table 3 and Table 8; β = -0.053 (95%-CI -0.101 to -0.005; p = 0.032)). The effect size of agoraphobia for adiponectin was lower than those of gender, obesity and underweight, but higher than those of overweight, hypertension and alcohol consumption. Levels of IL-6 (Table 3 and Table 6) and IL-1β (Table 3 and Table 5) did not significantly differ between groups. The composite score of proinflammatory markers (including CRP, IL-6 and TNF-α) at follow-up was higher (model 5: β = 0.578 (95%-CI 0.241–0.915; Table 3 and Table 9) among subjects suffering from lifetime agoraphobia at baseline. The severity of agoraphobia (mean GAF score ± SD: 53.9 ± 0.9 (range: 30–99)) was not significantly associated with the levels of the proinflammatory markers.

**Table 3:** Associations between lifetime agoraphobia status at baseline and inflammatory measures at follow-up, fully adjusted model.

	CRP^h^ (n = 2890)					Interleukin-1β^i^ (n = 2792)					Interleukin-6^h^ (n = 2792)					TNF-α^h^ (n = 2792)					Adiponectin^h^ (n = 2679)					Composite score^h^ ^,^ ^j^ (n = 2569)				
	Model 5					Model 5					Model 5					Model 5					Model 5					Model 5				
	β	95CI			p	OR	β			95CI	p	95CI			p	β	95CI			p	β	95CI			p	β	95CI			p
**Agoraphobia at baseline**	0.087	0.024	-	0.151	**0.007**	0.803	0.540	-	1.193	0.277	0.102	-0.038	-	0.241	0.153	0.089	0.003	-	0.174	**0.042**	-0.053	-0.101	-	-0.005	**0.032**	0.578	0.241	-	0.915	**0.001**
**Length of follow-up** ^**a**^ **, years**	-0.006	-0.038	-	0.025	0.686	1.052	0.856	-	1.294	0.629	-0.028	-0.099	-	0.044	0.449	-0.004	-0.047	-	0.040	0.876	0.069	0.043	-	0.095	**<0.001**	-0.122	-0.301	-	0.057	0.182
**Inflammatory markers at baseline** ^**b**^	0.097	0.089	-	0.105	**<0.001**	3.998	3.402	-	4.699	**<0.001**	0.003	0.002	-	0.003	**<0.001**	0.001	0.001	-	0.001	**<0.001**	0.011	0.010	-	0.013	**<0.001**	0.408	0.373	-	0.443	**<0.001**
**Age, years**	0.003	0.002	-	0.005	**<0.001**	0.987	0.978	-	0.997	**0.008**	-0.001	-0.005	-	0.002	0.522	0.003	0.001	-	0.005	**0.002**	0.004	0.003	-	0.005	**<0.001**	0.006	-0.002	-	0.014	0.164
**Male**	0.027	-0.004	-	0.058	0.090	1.104	0.909	-	1.340	0.318	-0.024	-0.092	-	0.045	0.499	-0.019	-0.061	-	0.023	0.370	0.146	0.122	-	0.170	**<0.001**	-0.018	-0.184	-	0.147	0.828
**Socioeconomic status** ^**c**^	-0.019	-0.030	-	-0.009	**<0.001**	1.019	0.954	-	1.087	0.583	-0.008	-0.031	-	0.015	0.487	-0.001	-0.015	-	0.013	0.897	-0.001	-0.009	-	0.007	0.836	-0.057	-0.113	-	-0.001	**0.045**
**Hypertension** ^**d**^	0.013	-0.021	-	0.046	0.460	1.205	0.977	-	1.486	0.082	-0.003	-0.076	-	0.071	0.942	0.014	-0.031	-	0.059	0.550	0.028	0.002	-	0.054	**0.033**	-0.004	-0.184	-	0.175	0.964
**Underweight**	-0.187	-0.286	-	-0.089	**<0.001**	1.629	0.843	-	3.148	0.146	-0.138	-0.361	-	0.085	0.225	-0.090	-0.226	-	0.047	0.199	0.116	0.037	-	0.195	**0.004**	-0.790	-1.322	-	-0.259	**0.004**
**Overweight**	0.091	0.060	-	0.122	**<0.001**	0.979	0.809	-	1.183	0.824	-0.026	-0.093	-	0.041	0.446	0.011	-0.030	-	0.052	0.605	-0.033	-0.057	-	-0.010	**0.006**	0.187	0.024	-	0.349	**0.025**
**Obesity**	0.125	0.078	-	0.173	**<0.001**	0.954	0.720	-	1.264	0.740	0.012	-0.087	-	0.111	0.806	0.050	-0.010	-	0.111	0.104	-0.069	-0.103	-	-0.034	**<0.001**	0.322	0.071	-	0.572	**0.012**
**Glucose, fasting state mmol/L**	-0.019	-0.033	-	-0.005	**0.008**	1.051	0.964	-	1.146	0.259	-0.013	-0.043	-	0.017	0.406	0.004	-0.015	-	0.022	0.701	-0.002	-0.013	-	0.009	0.706	-0.049	-0.125	-	0.027	0.209
**Total cholesterol/HDL-cholesterol ratio**	0.024	0.011	-	0.037	**<0.001**	0.960	0.885	-	1.042	0.327	0.040	0.011	-	0.068	**0.007**	0.017	-0.001	-	0.034	0.064	-0.036	-0.046	-	-0.026	**<0.001**	0.104	0.034	-	0.174	**0.004**
**Former/current smokers**	0.025	0.009	-	0.041	**0.003**	1.035	0.934	-	1.147	0.514	0.034	-0.002	-	0.070	0.063	0.015	-0.007	-	0.037	0.192	-0.003	-0.016	-	0.010	0.625	0.090	0.002	-	0.179	**0.046**
**Low alcohol consumption**	-0.038	-0.071	-	-0.005	**0.023**	1.054	0.856	-	1.298	0.623	-0.023	-0.096	-	0.050	0.535	-0.014	-0.058	-	0.031	0.549	0.011	-0.015	-	0.037	0.399	-0.070	-0.248	-	0.108	0.440
**Moderate alcohol consumption**	-0.047	-0.086	-	-0.008	**0.019**	1.020	0.797	-	1.306	0.874	-0.051	-0.137	-	0.036	0.253	-0.046	-0.099	-	0.007	0.088	0.018	-0.013	-	0.048	0.259	-0.247	-0.456	-	-0.037	**0.021**
**High alcohol consumption**	-0.008	-0.052	-	0.036	0.717	1.285	0.976	-	1.691	0.074	-0.041	-0.137	-	0.055	0.406	-0.017	-0.076	-	0.042	0.580	0.041	0.007	-	0.075	**0.019**	-0.135	-0.369	-	0.099	0.258
**Leisure physical activity** ^**e**^	-0.008	-0.034	-	0.018	0.543	1.006	0.852	-	1.188	0.946	0.024	-0.034	-	0.083	0.412	0.035	-0.001	-	0.070	0.058	-0.013	-0.034	-	0.007	0.208	0.079	-0.063	-	0.220	0.274
**Major depressive disorder**	0.002	-0.025	-	0.029	0.882	0.991	0.838	-	1.173	0.920	-0.061	-0.120	-	-0.002	**0.045**	-0.014	-0.050	-	0.022	0.449	-0.005	-0.026	-	0.016	0.639	-0.083	-0.227	-	0.060	0.256
**Other anxiety disorders** ^**f**^	-0.003	-0.036	-	0.029	0.839	1.130	0.921	-	1.386	0.242	-0.013	-0.084	-	0.059	0.729	0.005	-0.039	-	0.049	0.826	0.007	-0.018	-	0.032	0.592	-0.018	-0.191	-	0.156	0.842
**Drug dependence** ^**g**^	-0.001	-0.081	-	0.078	0.977	0.779	0.463	-	1.309	0.346	0.147	-0.036	-	0.331	0.115	0.115	0.002	-	0.227	**0.046**	0.011	-0.053	-	0.074	0.744	0.352	-0.084	-	0.789	0.114

Values for inflammatory measures are given as unadjusted log10-transformed means ± standard deviation, values for interleukin-1β are given as prevalence.

HDL, high-density lipoprotein; CRP, C-reactive protein; TNF, tumor necrosis factor; OR, odds ratio; 95CI, 95% confidence interval.

^a^ Duration between somatic evaluation at baseline and somatic evaluation at follow-up.

^b^ Model adjusted for the inflammatory marker at baseline corresponding to the specific inflammatory marker at follow-up.

^c^ A value of “3” represents an SES of III (middle class) on the Hollingshead Scale.

^d^ Systolic blood pressure ≥ 140 mmHg and/or diastolic blood pressure ≥ 90 mmHg.

^e^ Physically active at least or more than 20 minutes twice a week.

^f^ Generalized anxiety disorder, panic disorder, posttraumatic stress disorder, and/or social phobia.

^g^ Marijuana, cocaine, solvent, hallucinogen, stimulant, sedative or/and narcotic dependence.

^h^ Multiple regression with log10transformed cytokine or CRP or adiponectin.

^i^ Logistic regression with interleukin-1β concentration dichotomized at the median.

^j^ Composite score of inflammatory markers includes CRP, interleukin-6 and TNF-α. Only participants who had data available for all three of these markers were included in these analyses.

**Table 4:** Associations between lifetime agoraphobia status at baseline and inflammatory measures (C-reactive protein) at follow-up, serially adjusted for covariates.

	CRP^g^ (n = 2890)																								
	Model 1					Model 2					Model 3					Model 4					Model 5				
	β	95CI			p	β	95CI			p	β	95CI			p	β	95CI			p	β	95CI			p
**Agoraphobia at baseline**	0.098	0.034	-	0.162	**0.003**	0.093	0.029	-	0.156	**0.005**	0.089	0.026	-	0.152	**0.005**	0.086	0.024	-	0.149	**0.007**	0.087	0.024	-	0.151	**0.007**
**Length of follow-up** ^**a**^ **, years**	-0.004	-0.036	-	0.028	0.813	-0.003	-0.034	-	0.029	0.866	-0.006	-0.037	-	0.025	0.691	-0.007	-0.038	-	0.024	0.672	-0.006	-0.038	-	0.025	0.686
**CRP at baseline**	0.115	0.107	-	0.122	**<0.001**	0.110	0.102	-	0.118	**<0.001**	0.098	0.090	-	0.106	**<0.001**	0.097	0.089	-	0.105	**<0.001**	0.097	0.089	-	0.105	**<0.001**
**Age, years**						0.004	0.002	-	0.005	**<0.001**	0.003	0.001	-	0.004	**<0.001**	0.003	0.002	-	0.005	**<0.001**	0.003	0.002	-	0.005	**<0.001**
**Male**						-0.019	-0.046	-	0.007	0.145	0.027	-0.002	-	0.056	0.066	0.027	-0.003	-	0.058	0.078	0.027	-0.004	-	0.058	0.090
**Socioeconomic status** ^**b**^						-0.028	-0.039	-	-0.018	**<0.001**	-0.022	-0.032	-	-0.012	**<0.001**	-0.019	-0.029	-	-0.009	**<0.001**	-0.019	-0.030	-	-0.009	**<0.001**
**Hypertension** ^**c**^											0.014	-0.019	-	0.048	0.405	0.013	-0.021	-	0.046	0.460	0.013	-0.021	-	0.046	0.460
**Underweight**											-0.180	-0.279	-	-0.081	**<0.001**	-0.188	-0.286	-	-0.089	**<0.001**	-0.187	-0.286	-	-0.089	**<0.001**
**Overweight**											0.089	0.059	-	0.120	**<0.001**	0.091	0.060	-	0.121	**<0.001**	0.091	0.060	-	0.122	**<0.001**
**Obesity**											0.123	0.076	-	0.171	**<0.001**	0.126	0.078	-	0.173	**<0.001**	0.125	0.078	-	0.173	**<0.001**
**Glucose, fasting state mmol/L**											-0.018	-0.032	-	-0.004	**0.010**	-0.019	-0.033	-	-0.005	**0.008**	-0.019	-0.033	-	-0.005	**0.008**
**Total cholesterol/HDL-cholesterol ratio**											0.027	0.014	-	0.040	**<0.001**	0.024	0.011	-	0.037	**<0.001**	0.024	0.011	-	0.037	**<0.001**
**Former/current smokers**																0.025	0.009	-	0.041	**0.002**	0.025	0.009	-	0.041	**0.003**
**Low alcohol consumption**																-0.038	-0.071	-	-0.005	**0.023**	-0.038	-0.071	-	-0.005	**0.023**
**Moderate alcohol consumption**																-0.047	-0.086	-	-0.007	**0.020**	-0.047	-0.086	-	-0.008	**0.019**
**High alcohol consumption**																-0.008	-0.052	-	0.035	0.715	-0.008	-0.052	-	0.036	0.717
**Leisure physical activity** ^**d**^																-0.008	-0.034	-	0.018	0.553	-0.008	-0.034	-	0.018	0.543
**Major depressive disorder**																					0.002	-0.025	-	0.029	0.882
**Other anxiety disorders** ^**e**^																					-0.003	-0.036	-	0.029	0.839
**Drug dependence** ^**f**^																					-0.001	-0.081	-	0.078	0.977

Values for inflammatory measures are given as unadjusted log10-transformed means ± standard deviation, values for interleukin-1β are given as prevalence.

HDL, high-density lipoprotein; CRP, C-reactive protein; TNF, tumor necrosis factor; OR, odds ratio; 95CI, 95% confidence interval.

^a^ Duration between somatic evaluation at baseline and somatic evaluation at follow-up.

^b^ A value of “3” represents an SES of III (middle class) on the Hollingshead Scale.

^c^ Systolic blood pressure ≥ 140 mmHg and/or diastolic blood pressure ≥ 90 mmHg.

^d^ Physically active at least or more than 20 minutes twice a week.

^e^ Generalized anxiety disorder, panic disorder, posttraumatic stress disorder, and/or social phobia.

^f^ Marijuana, cocaine, solvent, hallucinogen, stimulant, sedative or/and narcotic dependence.

^g^ Multiple regression with log10-transformed cytokine or CRP or adiponectin.

^h^ Logistic regression with interleukin-1β concentration dichotomized at the median.

^i^ Composite score of inflammatory markers includes CRP, interleukin-6 and TNF-α. Only participants who had data available for all three of these markers were included in these analyses.

**Table 5:** Associations between lifetime agoraphobia status at baseline and inflammatory measures (interleukin-1β) at follow-up, serially adjusted for covariates.

	Interleukin-1β^h^ (n = 2792)																								
	Model 1					Model 2					Model 3					Model 4					Model 5				
	OR	95CI			p	OR	95CI			p	OR	95CI			p	OR	95CI			p	OR	95CI			p
**Agoraphobia at baseline**	0.840	0.571	-	1.236	0.376	0.830	0.563	-	1.224	0.347	0.821	0.556	-	1.213	0.322	0.829	0.561	-	1.226	0.347	0.803	0.540	-	1.193	0.277
**Length of follow-up** ^**a**^ **, years**	1.040	0.847	-	1.277	0.711	1.039	0.846	-	1.276	0.717	1.053	0.857	-	1.294	0.625	1.052	0.856	-	1.293	0.632	1.052	0.856	-	1.294	0.629
**Interleukin-1β at baseline**	3.944	3.362	-	4.627	**<0.001**	3.905	3.328	-	4.582	**<0.001**	3.950	3.364	-	4.639	**<0.001**	3.987	3.393	-	4.685	**<0.001**	3.998	3.402	-	4.699	**<0.001**
**Age, years**						0.990	0.981	-	0.999	**0.025**	0.987	0.978	-	0.997	**0.009**	0.987	0.978	-	0.997	**0.008**	0.987	0.978	-	0.997	**0.008**
**Male**						1.078	0.918	-	1.266	0.361	1.069	0.893	-	1.281	0.467	1.121	0.927	-	1.355	0.239	1.104	0.909	-	1.340	0.318
**Socioeconomic status** ^**b**^						1.016	0.954	-	1.082	0.625	1.017	0.954	-	1.085	0.603	1.018	0.953	-	1.086	0.598	1.019	0.954	-	1.087	0.583
**Hypertension** ^**c**^											1.225	0.995	-	1.508	0.056	1.205	0.977	-	1.486	0.081	1.205	0.977	-	1.486	0.082
**Underweight**											1.651	0.857	-	3.180	0.134	1.623	0.841	-	3.133	0.149	1.629	0.843	-	3.148	0.146
**Overweight**											0.980	0.811	-	1.184	0.832	0.983	0.813	-	1.188	0.858	0.979	0.809	-	1.183	0.824
**Obesity**											0.938	0.709	-	1.239	0.651	0.954	0.720	-	1.263	0.742	0.954	0.720	-	1.264	0.740
**Glucose, fasting state mmol/L**											1.053	0.966	-	1.148	0.238	1.050	0.963	-	1.145	0.268	1.051	0.964	-	1.146	0.259
**Total cholesterol/HDL-cholesterol ratio**											0.961	0.886	-	1.041	0.329	0.960	0.885	-	1.042	0.330	0.960	0.885	-	1.042	0.327
**Former/current smokers**																1.030	0.930	-	1.140	0.574	1.035	0.934	-	1.147	0.514
**Low alcohol consumption**																1.053	0.855	-	1.297	0.626	1.054	0.856	-	1.298	0.623
**Moderate alcohol consumption**																1.020	0.797	-	1.306	0.873	1.020	0.797	-	1.306	0.874
**High alcohol consumption**																1.284	0.976	-	1.690	0.075	1.285	0.976	-	1.691	0.074
**Leisure physical activity** ^**d**^																1.008	0.854	-	1.190	0.927	1.006	0.852	-	1.188	0.946
**Major depressive disorder**																					0.991	0.838	-	1.173	0.920
**Other anxiety disorders** ^**e**^																					1.130	0.921	-	1.386	0.242
**Drug dependence** ^**f**^					** **					** **					** **						0.779	0.463	-	1.309	0.346

Values for inflammatory measures are given as unadjusted log10-transformed means ± standard deviation, values for interleukin-1β are given as prevalence.

HDL, high-density lipoprotein; CRP, C-reactive protein; TNF, tumor necrosis factor; OR, odds ratio; 95CI, 95% confidence interval.

^a^ Duration between somatic evaluation at baseline and somatic evaluation at follow-up.

^b^ A value of “3” represents an SES of III (middle class) on the Hollingshead Scale.

^c^ Systolic blood pressure ≥ 140 mmHg and/or diastolic blood pressure ≥ 90 mmHg.

^d^ Physically active at least or more than 20 minutes twice a week.

^e^ Generalized anxiety disorder, panic disorder, posttraumatic stress disorder, and/or social phobia.

^f^ Marijuana, cocaine, solvent, hallucinogen, stimulant, sedative or/and narcotic dependence.

^g^ Multiple regression with log10 transformed cytokine or CRP or adiponectin.

^h^ Logistic regression with interleukin-1β concentration dichotomized at the median.

^i^ Composite score of inflammatory markers includes CRP, interleukin-6 and TNF-α. Only participants who had data available for all three of these markers were included in these analyses.

**Table 6:** Associations between lifetime agoraphobia status at baseline and inflammatory measures (interleukin-6) at follow-up, serially adjusted for covariates.

	Interleukin-6^g^ (n = 2792)																								
	Model 1					Model 2					Model 3					Model 4					Model 5				
	β	95CI			p	β	95CI			p	β	95CI			p	β	95CI			p	β	95CI			p
**Agoraphobia at baseline**	0.093	-0.044	-	0.230	0.184	0.105	-0.033	-	0.242	0.135	0.101	-0.037	-	0.238	0.152	0.095	-0.043	-	0.232	0.178	0.102	-0.038	-	0.241	0.153
**Length of follow-up** ^**a**^ **, years**	-0.012	-0.084	-	0.059	0.735	-0.016	-0.087	-	0.056	0.667	-0.023	-0.094	-	0.049	0.534	-0.024	-0.095	-	0.048	0.520	-0.028	-0.099	-	0.044	0.449
**Interleukin-6 at baseline**	0.003	0.002	-	0.003	**<0.001**	0.003	0.002	-	0.003	**<0.001**	0.003	0.002	-	0.003	**<0.001**	0.003	0.002	-	0.003	**<0.001**	0.003	0.002	-	0.003	**<0.001**
**Age, years**						-0.001	-0.004	-	0.002	0.495	-0.001	-0.004	-	0.002	0.520	-0.001	-0.004	-	0.002	0.543	-0.001	-0.005	-	0.002	0.522
**Male**						-0.061	-0.118	-	-0.005	**0.034**	-0.033	-0.097	-	0.031	0.309	-0.041	-0.108	-	0.026	0.228	-0.024	-0.092	-	0.045	0.499
**Socioeconomic status** ^**b**^						-0.012	-0.034	-	0.010	0.286	-0.011	-0.033	-	0.012	0.363	-0.009	-0.032	-	0.014	0.444	-0.008	-0.031	-	0.015	0.487
**Hypertension** ^**c**^											-0.006	-0.080	-	0.067	0.867	-0.003	-0.077	-	0.071	0.938	-0.003	-0.076	-	0.071	0.942
**Underweight**											-0.121	-0.344	-	0.102	0.288	-0.134	-0.357	-	0.090	0.241	-0.138	-0.361	-	0.085	0.225
**Overweight**											-0.031	-0.097	-	0.036	0.365	-0.027	-0.094	-	0.040	0.424	-0.026	-0.093	-	0.041	0.446
**Obesity**											0.004	-0.094	-	0.102	0.936	0.011	-0.088	-	0.109	0.835	0.012	-0.087	-	0.111	0.806
**Glucose, fasting state mmol/L**											-0.013	-0.043	-	0.018	0.412	-0.012	-0.043	-	0.018	0.426	-0.013	-0.043	-	0.017	0.406
**Total cholesterol/HDL-cholesterol ratio**											0.042	0.014	-	0.071	**0.003**	0.039	0.010	-	0.068	**0.008**	0.040	0.011	-	0.068	**0.007**
**Former/current smokers**																0.035	-0.001	-	0.071	0.055	0.034	-0.002	-	0.070	0.063
**Low alcohol consumption**																-0.025	-0.098	-	0.048	0.501	-0.023	-0.096	-	0.050	0.535
**Moderate alcohol consumption**																-0.048	-0.134	-	0.039	0.282	-0.051	-0.137	-	0.036	0.253
**High alcohol consumption**																-0.039	-0.135	-	0.058	0.429	-0.041	-0.137	-	0.055	0.406
**Leisure physical activity** ^**d**^																0.024	-0.034	-	0.083	0.417	0.024	-0.034	-	0.083	0.412
**Major depressive disorder**																					-0.061	-0.120	-	-0.002	**0.045**
**Other anxiety disorders** ^**e**^																					-0.013	-0.084	-	0.059	0.729
**Drug dependence** ^**f**^					** **					** **					** **						0.147	-0.036	-	0.331	0.115

Values for inflammatory measures are given as unadjusted log10-transformed means ± standard deviation, values for interleukin-1β are given as prevalence.

HDL, high-density lipoprotein; CRP, C-reactive protein; TNF, tumor necrosis factor; OR, odds ratio; 95CI, 95% confidence interval.

^a^ Duration between somatic evaluation at baseline and somatic evaluation at follow-up.

^b^ A value of “3” represents an SES of III (middle class) on the Hollingshead Scale.

^c^ Systolic blood pressure ≥ 140 mmHg and/or diastolic blood pressure ≥ 90 mmHg.

^d^ Physically active at least or more than 20 minutes twice a week.

^e^ Generalized anxiety disorder, panic disorder, posttraumatic stress disorder, and/or social phobia.

^f^ Marijuana, cocaine, solvent, hallucinogen, stimulant, sedative or/and narcotic dependence.

^g^ Multiple regression with log10 transformed cytokine or CRP or adiponectin.

^h^ Logistic regression with interleukin-1β concentration dichotomized at the median.

^i^ Composite score of inflammatory markers includes CRP, interleukin-6 and TNF-α. Only participants who had data available for all three of these markers were included in these analyses.

**Table 7:** Associations between lifetime agoraphobia status at baseline and inflammatory measures (tumor necrosis factor-α) at follow-up, serially adjusted for covariates.

	TNF-α^g^ (n = 2792)																								
	Model 1					Model 2					Model 3					Model 4					Model 5				
	β	95CI			p	β	95CI			p	β	95CI			p	β	95CI			p	β	95CI			p
**Agoraphobia at baseline**	0.086	0.002	-	0.170	**0.044**	0.094	0.010	-	0.178	**0.029**	0.092	0.008	-	0.176	**0.033**	0.091	0.006	-	0.175	**0.035**	0.089	0.003	-	0.174	**0.042**
**Length of follow-up** ^**a**^ **, years**	-0.001	-0.045	-	0.043	0.974	0.000	-0.044	-	0.044	0.999	-0.003	-0.046	-	0.041	0.908	-0.003	-0.047	-	0.041	0.896	-0.004	-0.047	-	0.040	0.876
**TNF-α at baseline**	0.001	0.001	-	0.001	**<0.001**	0.001	0.001	-	0.001	**<0.001**	0.001	0.001	-	0.001	**<0.001**	0.001	0.001	-	0.001	**<0.001**	0.001	0.001	-	0.001	**<0.001**
**Age, years**						0.004	0.002	-	0.006	**<0.001**	0.003	0.001	-	0.005	**0.002**	0.003	0.001	-	0.005	**0.003**	0.003	0.001	-	0.005	**0.002**
**Male**						-0.042	-0.077	-	-0.007	**0.018**	-0.019	-0.058	-	0.020	0.340	-0.024	-0.065	-	0.017	0.245	-0.019	-0.061	-	0.023	0.370
**Socioeconomic status** ^**b**^						-0.005	-0.019	-	0.009	0.467	-0.002	-0.015	-	0.012	0.832	-0.001	-0.015	-	0.013	0.871	-0.001	-0.015	-	0.013	0.897
**Hypertension** ^**c**^											0.011	-0.034	-	0.056	0.637	0.013	-0.032	-	0.058	0.567	0.014	-0.031	-	0.059	0.550
**Underweight**											-0.078	-0.214	-	0.059	0.266	-0.087	-0.224	-	0.049	0.210	-0.090	-0.226	-	0.047	0.199
**Overweight**											0.006	-0.035	-	0.047	0.775	0.009	-0.032	-	0.050	0.659	0.011	-0.030	-	0.052	0.605
**Obesity**											0.042	-0.019	-	0.102	0.176	0.048	-0.013	-	0.108	0.122	0.050	-0.010	-	0.111	0.104
**Glucose, fasting state mmol/L**											0.003	-0.015	-	0.022	0.720	0.004	-0.015	-	0.023	0.671	0.004	-0.015	-	0.022	0.701
**Total cholesterol/HDL-cholesterol ratio**											0.018	0.000	-	0.035	**0.046**	0.017	-0.001	-	0.034	0.064	0.017	-0.001	-	0.034	0.064
**Former/current smokers**																0.017	-0.005	-	0.039	0.131	0.015	-0.007	-	0.037	0.192
**Low alcohol consumption**																-0.015	-0.060	-	0.030	0.517	-0.014	-0.058	-	0.031	0.549
**Moderate alcohol consumption**																-0.045	-0.098	-	0.008	0.098	-0.046	-0.099	-	0.007	0.088
**High alcohol consumption**																-0.017	-0.076	-	0.042	0.581	-0.017	-0.076	-	0.042	0.580
**Leisure physical activity** ^**d**^																0.034	-0.002	-	0.070	0.063	0.035	-0.001	-	0.070	0.058
**Major depressive disorder**																					-0.014	-0.050	-	0.022	0.449
**Other anxiety disorders** ^**e**^																					0.005	-0.039	-	0.049	0.826
**Drug dependence** ^**f**^																					0.115	0.002	-	0.227	**0.046**

Values for inflammatory measures are given as unadjusted log10-transformed means ± standard deviation, values for interleukin-1β are given as prevalence.

HDL, high-density lipoprotein; CRP, C-reactive protein; TNF, tumor necrosis factor; OR, odds ratio; 95CI, 95% confidence interval.

^a^ Duration between somatic evaluation at baseline and somatic evaluation at follow-up.

^b^ A value of “3” represents an SES of III (middle class) on the Hollingshead Scale.

^c^ Systolic blood pressure ≥ 140 mmHg and/or diastolic blood pressure ≥ 90 mmHg.

^d^ Physically active at least or more than 20 minutes twice a week.

^e^ Generalized anxiety disorder, panic disorder, posttraumatic stress disorder, and/or social phobia.

^f^ Marijuana, cocaine, solvent, hallucinogen, stimulant, sedative or/and narcotic dependence.

^g^ Multiple regression with log10 transformed cytokine or CRP or adiponectin.

^h^ Logistic regression with interleukin-1β concentration dichotomized at the median.

^i^ Composite score of inflammatory markers includes CRP, interleukin-6 and TNF-α. Only participants who had data available for all three of these markers were included in these analyses.

**Table 8:** Associations between lifetime agoraphobia status at baseline and inflammatory measures (adiponectin) at follow-up, serially adjusted for covariates.

	Adiponectin^g^ (n = 2679)																								
	Model 1					Model 2					Model 3					Model 4					Model 5				
	β	95CI			p	β	95CI			p	β	95CI			p	β	95CI			p	β	95CI			p
**Agoraphobia at baseline**	-0.015	-0.066	-	0.036	0.562	-0.056	-0.105	-	-0.008	**0.023**	-0.053	-0.100	-	-0.005	**0.030**	-0.051	-0.099	-	-0.003	**0.036**	-0.053	-0.101	-	-0.005	**0.032**
**Length of follow-up** ^**a**^ **, years**	0.061	0.034	-	0.089	**<0.001**	0.065	0.039	-	0.091	**<0.001**	0.069	0.044	-	0.095	**<0.001**	0.069	0.044	-	0.095	**<0.001**	0.069	0.043	-	0.095	**<0.001**
**Adiponectin at baseline**	0.017	0.015	-	0.018	**<0.001**	0.013	0.011	-	0.014	**<0.001**	0.011	0.010	-	0.013	**<0.001**	0.011	0.010	-	0.013	**<0.001**	0.011	0.010	-	0.013	**<0.001**
**Age, years**						0.003	0.002	-	0.004	**<0.001**	0.004	0.003	-	0.005	**<0.001**	0.004	0.003	-	0.005	**<0.001**	0.004	0.003	-	0.005	**<0.001**
**Male**						0.170	0.148	-	0.191	**<0.001**	0.136	0.114	-	0.159	**<0.001**	0.145	0.121	-	0.169	**<0.001**	0.146	0.122	-	0.170	**<0.001**
**Socioeconomic status** ^**b**^						0.005	-0.003	-	0.013	0.260	-0.001	-0.009	-	0.007	0.806	-0.001	-0.009	-	0.007	0.808	-0.001	-0.009	-	0.007	0.836
**Hypertension** ^**c**^											0.032	0.006	-	0.058	**0.016**	0.028	0.002	-	0.054	**0.034**	0.028	0.002	-	0.054	**0.033**
**Underweight**											0.115	0.036	-	0.194	**0.004**	0.116	0.037	-	0.195	**0.004**	0.116	0.037	-	0.195	**0.004**
**Overweight**											-0.032	-0.056	-	-0.009	**0.007**	-0.033	-0.057	-	-0.010	**0.006**	-0.033	-0.057	-	-0.010	**0.006**
**Obesity**											-0.069	-0.103	-	-0.034	**<0.001**	-0.069	-0.104	-	-0.034	**<0.001**	-0.069	-0.103	-	-0.034	**<0.001**
**Glucose, fasting state mmol/L**											-0.001	-0.012	-	0.009	0.812	-0.002	-0.013	-	0.009	0.707	-0.002	-0.013	-	0.009	0.706
**Total cholesterol/HDL-cholesterol ratio**											-0.036	-0.046	-	-0.026	**<0.001**	-0.036	-0.046	-	-0.026	**<0.001**	-0.036	-0.046	-	-0.026	**<0.001**
**Former/current smokers**																-0.003	-0.016	-	0.010	0.626	-0.003	-0.016	-	0.010	0.625
**Low alcohol consumption**																0.011	-0.015	-	0.037	0.406	0.011	-0.015	-	0.037	0.399
**Moderate alcohol consumption**																0.018	-0.013	-	0.048	0.254	0.018	-0.013	-	0.048	0.259
**High alcohol consumption**																0.041	0.007	-	0.075	**0.019**	0.041	0.007	-	0.075	**0.019**
**Leisure physical activity** ^**d**^																-0.013	-0.034	-	0.007	0.205	-0.013	-0.034	-	0.007	0.208
**Major depressive disorder**																					-0.005	-0.026	-	0.016	0.639
**Other anxiety disorders** ^**e**^																					0.007	-0.018	-	0.032	0.592
**Drug dependence** ^**f**^																					0.011	-0.053	-	0.074	0.744

Values for inflammatory measures are given as unadjusted log10-transformed means ± standard deviation, values for interleukin-1β are given as prevalence.

HDL, high-density lipoprotein; CRP, C-reactive protein; TNF, tumor necrosis factor; OR, odds ratio; 95CI, 95% confidence interval.

^a^ Duration between somatic evaluation at baseline and somatic evaluation at follow-up.

^b^ A value of “3” represents an SES of III (middle class) on the Hollingshead Scale.

^c^ Systolic blood pressure ≥ 140 mmHg and/or diastolic blood pressure ≥ 90 mmHg.

^d^ Physically active at least or more than 20 minutes twice a week.

^e^ Generalized anxiety disorder, panic disorder, posttraumatic stress disorder, and/or social phobia.

^f^ Marijuana, cocaine, solvent, hallucinogen, stimulant, sedative or/and narcotic dependence.

^g^ Multiple regression with log10 transformed cytokine or CRP or adiponectin.

^h^ Logistic regression with interleukin-1β concentration dichotomized at the median.

^i^ Composite score of inflammatory markers includes CRP, interleukin-6 and TNF-α. Only participants who had data available for all three of these markers were included in these analyses.

**Table 9:** Associations between lifetime agoraphobia status at baseline and inflammatory measures (composite score) at follow-up, serially adjusted for covariates.

	Composite score^g^ ^,^ ^i^ (n = 2569)																								
	Model 1					Model 2					Model 3					Model 4					Model 5				
	β	95CI			p	β	95CI			p	β	95CI			p	β	95CI			p	β	95CI			p
**Agoraphobia at baseline**	0.573	0.240	-	0.906	**0.001**	0.591	0.256	-	0.926	**0.001**	0.584	0.250	-	0.917	**0.001**	0.568	0.235	-	0.902	**0.001**	0.578	0.241	-	0.915	**0.001**
**Length of follow-up** ^**a**^ **, years**	-0.091	-0.272	-	0.089	0.322	-0.096	-0.276	-	0.084	0.297	-0.116	-0.295	-	0.064	0.206	-0.118	-0.298	-	0.061	0.195	-0.122	-0.301	-	0.057	0.182
**Composite score at baseline**	0.444	0.410	-	0.478	**<0.001**	0.434	0.400	-	0.469	**<0.001**	0.414	0.379	-	0.448	**<0.001**	0.410	0.375	-	0.445	**<0.001**	0.408	0.373	-	0.443	**<0.001**
**Age, years**						0.007	-0.001	-	0.015	0.081	0.005	-0.003	-	0.013	0.203	0.006	-0.003	-	0.014	0.179	0.006	-0.002	-	0.014	0.164
**Male**						-0.146	-0.285	-	-0.008	**0.039**	-0.009	-0.164	-	0.145	0.906	-0.045	-0.207	-	0.117	0.586	-0.018	-0.184	-	0.147	0.828
**Socioeconomic status** ^**b**^						-0.081	-0.136	-	-0.026	**0.004**	-0.064	-0.119	-	-0.008	**0.024**	-0.058	-0.114	-	-0.002	**0.042**	-0.057	-0.113	-	-0.001	**0.045**
**Hypertension** ^**c**^											-0.017	-0.196	-	0.162	0.852	-0.005	-0.185	-	0.174	0.953	-0.004	-0.184	-	0.175	0.964
**Underweight**											-0.740	-1.272	-	-0.209	**0.006**	-0.784	-1.316	-	-0.253	**0.004**	-0.790	-1.322	-	-0.259	**0.004**
**Overweight**											0.171	0.008	-	0.333	**0.040**	0.183	0.020	-	0.346	**0.028**	0.187	0.024	-	0.349	**0.025**
**Obesity**											0.301	0.051	-	0.551	**0.018**	0.316	0.065	-	0.566	**0.014**	0.322	0.071	-	0.572	**0.012**
**Glucose, fasting state mmol/L**											-0.051	-0.127	-	0.026	0.194	-0.048	-0.125	-	0.028	0.214	-0.049	-0.125	-	0.027	0.209
**Total cholesterol/HDL-cholesterol ratio**											0.112	0.043	-	0.182	**0.002**	0.102	0.032	-	0.173	**0.004**	0.104	0.034	-	0.174	**0.004**
**Former/current smokers**																0.097	0.009	-	0.185	**0.030**	0.090	0.002	-	0.179	**0.046**
**Low alcohol consumption**																-0.075	-0.253	-	0.103	0.409	-0.070	-0.248	-	0.108	0.440
**Moderate alcohol consumption**																-0.238	-0.448	-	-0.028	**0.026**	-0.247	-0.456	-	-0.037	**0.021**
**High alcohol consumption**																-0.133	-0.368	-	0.101	0.264	-0.135	-0.369	-	0.099	0.258
**Leisure physical activity** ^**d**^																0.078	-0.063	-	0.220	0.278	0.079	-0.063	-	0.220	0.274
**Major depressive disorder**																					-0.083	-0.227	-	0.060	0.256
**Other anxiety disorders** ^**e**^																					-0.018	-0.191	-	0.156	0.842
**Drug dependence** ^**f**^																					0.352	-0.084	-	0.789	0.114

Values for inflammatory measures are given as unadjusted log10-transformed means ± standard deviation, values for interleukin-1β are given as prevalence.

HDL, high-density lipoprotein; CRP, C-reactive protein; TNF, tumor necrosis factor; OR, odds ratio; 95CI, 95% confidence interval.

^a^ Duration between somatic evaluation at baseline and somatic evaluation at follow-up.

^b^ A value of “3” represents an SES of III (middle class) on the Hollingshead Scale.

^c^ Systolic blood pressure ≥ 140 mmHg and/or diastolic blood pressure ≥ 90 mmHg.

^d^ Physically active at least or more than 20 minutes twice a week.

^e^ Generalized anxiety disorder, panic disorder, posttraumatic stress disorder, and/or social phobia.

^f^ Marijuana, cocaine, solvent, hallucinogen, stimulant, sedative or/and narcotic dependence.

^g^ Multiple regression with log10 transformed cytokine or CRP or adiponectin.

^h^ Logistic regression with interleukin-1β concentration dichotomized at the median.

^i^ Composite score of inflammatory markers includes CRP, interleukin-6 and TNF-α. Only participants who had data available for all three of these markers were included in these analyses.

## Discussion

In this population-based sample of 2890 Swiss adults, individuals with agoraphobia, relative to their non-agoraphobic counterparts, showed a prospective increase in levels of circulating biomarkers indicating a low-grade inflammatory state over time. Especially, levels of CRP, but also of TNF-α increased over time, while the level of cardio-protective adiponectin significantly decreased from baseline to follow-up. The direction of the association between agoraphobia and IL-6 was also as hypothesized direction, although the association was not significantly, suggesting insufficient statistical power. Interestingly, the proinflammatory biomarkers and the adipo(cyto)kine adiponectin showed no difference between the two groups at baseline. The intriguing finding of discrepancies between the results from baseline and follow-up were not explained by the serial adjustments for potential confounders (including the length of follow-up). However, this might support the notion that the agoraphobic individuals in our sample are more vulnerable to a progressive immune function dysregulation over time. This might become rather evident at the age range examined (35–67 years).

Our findings of an association between agoraphobia and low-grade inflammation concur with studies on individuals with PTSD [2–5, 7, 11]. Whether psychobiological mechanisms, including HPA axis dysfunction, which have been identified to possibly link PTSD with inflammation, also apply to agoraphobics needs to be investigated further in carefully planned mechanistic studies.

To our knowledge this is the first study that examined the prospective associations between agoraphobia and changes in proinflammatory biomarkers in a large population-based sample. The strength of the study is the use of both a thorough biological evaluation and a comprehensive psychiatric assessment which allowed us to collect data on environmental and health-related risk factors as well as on major psychiatric diseases. The overall prevalence of agoraphobia in this study was 4.3%, which is situated within the lower bound of prevalences reported in other studies, ranging from 1% to 22% [18]. We found a striking gender difference in the prevalence of agoraphobia with almost 4 out of 5 participants of the agoraphobic group being female (81%). This prevalence estimate is similar to that of another study reporting a women to men ratio of 4:1 for agoraphobia [43].

The PsyCoLaus study was designed to better understand the relationship between psychiatric disorders and cardiovascular diseases (CVD) [23], including the potential mechanisms involved in this link, such as chronic low-grade inflammation. Meta-analyses suggest that the prospective risk of incident coronary heart disease (CHD) is increased in individuals with an anxiety disorder, including phobic anxiety [44], as well as in those with elevated levels of inflammatory measures, including CRP and TNF-α [45, 46]. Enhanced inflammation is a key process in atherosclerosis, which, for instance, via crosstalking with the endothelium and coagulation system promotes endothelial dysfunction and prothrombotic changes that critically contribute to atherosclerosis progression and ultimately acute coronary syndromes [47, 48]. Abundant biobehavioral research strongly suggests that psychosocial risk factors, including psychiatric disorders may affect all of the biological processes that lead from initiation to overt manifestation of atherothrombotic diseases, a process that may take many decades to become clinically apparent [49, 50]. Taken together, this research together with findings from our study may imply that a chronic low-grade inflammatory state in agoraphobia contributes to atherothrombotic CVD in patients with agoraphobia independent of sociodemographic, life style and physical CVRFs, and other major psychiatric diseases.

This study should be placed into the context of at least two notable limitations. First, as a consequence of avoidance behaviors (e.g., travel to the study site), some of the most severe cases of agoraphobia may not have been included in our sample. This may limit the generalization of our findings to all patients with agoraphobia, and particularly to those who are referred to a mental health care setting for treatment. However, as persons with severe agoraphobia could potentially show even higher levels of inflammatory markers, our results are likely to entail a conservative estimate. Second, the gap between the psychiatric and somatic assessments was about one year, which might have diluted temporal relationships between agoraphobia and inflammation, thereby potentially explaining why IL-1β and IL-6 levels showed no significant associations. Also, this between assessment gap entailed the risk that some individuals attributed to agoraphobia had produced cytokine levels that preceded the onset of the disorder. In this regard, it was shown that anxiety symptoms are part of cytokine-induced sickness behavior resulting from peripheral inflammation signaling the brain to initiate adaptive behaviors in various states of immune activation [51]. However, full-blown agoraphobic symptoms are not usually viewed as typical for sickness behavior and, moreover, the associations in our study showed high robustness, even after adjustment for a set of important CVRFs.

To sum up, our study demonstrates that agoraphobia predicted an increase in low-grade systematic inflammation over time in comparison to a control group without agoraphobia. This finding is clinically relevant, suggesting that agoraphobia might exaggerate inflammation activity which, further downstream, might result in vascular pathology and ultimately atherosclerotic vascular disease. However, longitudinal studies with CVD endpoints are needed to confirm this possibility. Future studies should also investigate which signs and symptoms of agoraphobia show a particularly strong association with chronic low-grade inflammation.

## References

[1] Hamer M, Gibson EL, Vuononvirta R, Williams E, Steptoe A. Inflammatory and hemostatic responses to repeated mental stress: individual stability and habituation over time. Brain Behav Immun. 2006;20: 456–459. 16488574 10.1016/j.bbi.2006.01.001

[2] von Känel R, Hepp U, Kraemer B, Traber R, Keel M, Mica L, et al Evidence for low-grade systemic proinflammatory activity in patients with posttraumatic stress disorder. J Psychiatr Res. 2007;41: 744–752. 16901505 10.1016/j.jpsychires.2006.06.009

[3] Gill JM, Saligan L, Woods S, Page G. PTSD is associated with an excess of inflammatory immune activities. Perspect Psychiatr Care. 2009;5: 262–277. 10.1111/j.1744-6163.2009.00229.x 19780999

[4] Pace TW, Heim CM. A short review on the psychoneuroimmunology of posttraumatic stress disorder: from risk factors to medical comorbidities. Brain Behav Immun. 2011;25: 6–13. 10.1016/j.bbi.2010.10.003 Epub 2010 Oct 8. Review. 20934505

[5] van Zuiden M, Kavelaars A, Geuze E, Olff M, Heijnen CJ. Predicting PTSD: pre-existing vulnerabilities in glucocorticoid-signaling and implications for preventive interventions. Brain Behav Immun. 2013;30: 12–21. 10.1016/j.bbi.2012.08.015 Epub 2012 Sep 5. Review. 22981834

[6] Sternberg EM. Neural regulation of innate immunity: A coordinated nonspecific host response to pathogens. Nature Reviews Immunology. 2006;6: 318–328. 16557263 10.1038/nri1810 PMC1783839

[7] Pervanidou P, Kolaitis G, Charitaki S, Margeli A, Ferentinos S, Bakoula C, et al Elevated morning serum interleukin (IL)-6 or evening salivary cortisol concentrations predict posttraumatic stress disorder in children and adolescents six months after a motor vehicle accident. Psychoneuroendocrinology. 2007;32: 991–999. 17825995 10.1016/j.psyneuen.2007.07.001

[8] Raison CL, Miller AH. When not enough is too much: The role of insufficient glucocorticoid signaling in the pathophysiology of stress-related disorders. American Journal of Psychiatry. 2003;160: 1554–1565. 12944327 10.1176/appi.ajp.160.9.1554

[9] Elenkov IJ, Tezzoni DG, Daly A, Harris AG, Chrousos GP. Cytokine dysregulation, inflammation and well-being. Neuroimmunomodulation. 2005;12: 255–269. 16166805 10.1159/000087104

[10] McEwen BS. Physiology and neurobiology of stress and adaptation: Central role of the brain. Physiological Reviews. 2007;87: 873–904. 17615391 10.1152/physrev.00041.2006

[11] Eraly SA, Nievergelt CM, Maihofer AX, Barkauskas DA, Biswas N, Agorastos A, et al Assessment of plasma C-reactive protein as a biomarker of posttraumatic stress disorder risk. JAMA Psychiatry. 2014;71: 423–431. 10.1001/jamapsychiatry.2013.4374 24576974 PMC4032578

[12] de Vries GJ, Olff M. The lifetime prevalence of traumatic events and posttraumatic stress disorder in the Netherlands. J Trauma Stress. 2009;22: 259–267. 10.1002/jts.20429 19645050

[13] Hoge EA, Brandstetter K, Moshier S, Pollack MH, Wong KK, Simon NM. Broad spectrum of cytokine abnormalities in panic disorder and posttraumatic stress disorder. Depress Anxiety. 2009;26: 447–455. 10.1002/da.20564 19319993

[14] Bankier B, Barajas J, Martinez-Rumayor A, Januzzi JL. Association between C-reactive protein and generalized anxiety disorder in stable coronary heart disease patients. Eur Heart J. 2008;29: 2212–2217. 10.1093/eurheartj/ehn326 18603621

[15] Buist-Bouwman MA, De Graaf R, Vollebergh WA, Alonso J, Bruffaerts R, Ormel J. Functional disability of mental disorders and comparison with physical disorders: a study among the general population of six European countries. Acta Psychiatr Scand. 2006;113: 492–500. 16677226 10.1111/j.1600-0447.2005.00684.x

[16] Emmelkamp PMG, Wittchen HU. Specific phobias In: Andrews G, Charney DS, Sirovatka PJ, Regier DA, editors. Stress-Induced and Fear Circuitry Disorders Refining the Research Agenda for DSM-V. Arlington, VA: American Psychiatric Publishing; 2009 pp. 77–101.

[17] American Psychiatric Association. Diagnostic and Statistical Manual of Mental Disorders Text Revision. 4th ed. Arlington, VA: American Psychiatric Publishing; 2000.

[18] Wittchen HU, Gloster AT, Beesdo-Baum K, Fava GA, Craske MG. Agoraphobia: a review of the diagnostic classificatory position and criteria. Depress Anxiety. 2010;27: 113–133. 10.1002/da.20646 20143426

[19] Surman OS, Williams J, Sheehan DV, Strom TB, Jones KJ, Coleman J. Immunological response to stress in agoraphobia and panic attacks. Biol Psychiatr. 1986;21: 768–774. 3524696 10.1016/0006-3223(86)90242-8

[20] Vogelzangs N, Beekman AT, de Jonge P, Penninx BW. Anxiety disorders and inflammation in a large adult cohort. Transl Psychiatry. 2013;3: e249 10.1038/tp.2013.27 23612048 PMC3641413

[21] Van de Voorde J, Pauwels B, Boydens C, Decaluwé K. Adipocytokines in relation to cardiovascular disease. Metabolism. 2013;62: 1513–1521. 10.1016/j.metabol.2013.06.004 23866981

[22] Firmann M, Mayor V, Vidal PM, Bochud M, Pécoud A, Hayoz D, et al The CoLaus study: a population-based study to investigate the epidemiology and genetic determinants of cardiovascular risk factors and metabolic syndrome. BMC Cardiovasc Disord. 2008;8: 6 10.1186/1471-2261-8-6 18366642 PMC2311269

[23] Preisig M, Waeber G, Vollenweider P, Bovet P, Rothen S, Vandeleur C, et al The PsyCoLaus study: methodology and characteristics of the sample of a population-based survey on psychiatric disorders and their association with genetic and cardiovascular risk factors. BMC Psychiatry. 2009;9: 9 10.1186/1471-244X-9-9 19292899 PMC2667506

[24] Goldberg DP. The detection of psychiatric illness by questionnaire Oxford, Great Britain: Oxford University Press; 1972.

[25] Bettschart W, Bolognini M. Questionnaire de santé, GHQ-12 [Health Questionnaire, GHQ-12] In: Guelfi JD, editor. L'évaluation clinique standardisée en psychiatrie Tome I [Standardized clinical evaluation in psychiatry Volume 1]. Boulogne, France: Editions Médicales Pierre Fabre; 1996.

[26] Nurnberger JI Jr, Blehar MC, Kaufmann CA, York-Cooler C, Simpson SG, Harkavy-Friedman J, et al Diagnostic interview for genetic studies. Rationale, unique features, and training. NIMH Genetics Initiative. Arch Gen Psychiatry. 1994;51: 849–859; discussion 863–864. 7944874 10.1001/archpsyc.1994.03950110009002

[27] Leboyer M, Barbe T, Gorwood P, Teherani M, Allilaire JF, Preisig M, et al Interview diagnostique pour les études génétiques Paris, France: INSERM; 1995.

[28] Preisig M, Fenton BT, Matthey ML, Berney A, Ferrero F. Diagnostic interview for genetic studies (DIGS): interrater and test-retest reliability of the French version. Eur Arch Psychiatry Clin Neurosci. 1999;249: 174–179. 10449592 10.1007/s004060050084

[29] Berney A, Preisig M, Matthey ML, Ferrero F, Fenton BT. Diagnostic interview for genetic studies (DIGS): interrater and test-retest reliability of alcohol and drug diagnoses. Drug Alcohol Depend. 2002;65: 149–158. 11772476 10.1016/s0376-8716(01)00156-9

[30] Rougemont-Buecking A, Rothen S, Jeanprêtre N, Lustenberger Y, Vandeleur CL, Ferrero F, et al Inter-informant agreement on diagnoses and prevalence estimates of anxiety disorders: direct interview versus family history method. Psychiatry Res. 2008;157: 211–223. 17881063 10.1016/j.psychres.2006.04.022

[31] Flower L, Ahuja RH, Humphries SE, Mohamed-Ali V. Effects of sample handling on the stability of interleukin 6, tumour necrosis factor-alpha and leptin. Cytokine. 2000;12: 1712–1716. 11052823 10.1006/cyto.2000.0764

[32] Skeppholm M, Wallén NH, Blombäck M, Kallner A. Can both EDTA and citrate plasma samples be used in measurements of fibrinogen and C-reactive protein concentrations? Clin Chem Lab Med. 2008;46: 1175–1179. 10.1515/CCLM.2008.219 18605954

[33] Vignali DA. Multiplexed particle-based flow cytometric assays. J Immunol Methods. 2000;243: 243–255. 10986418 10.1016/s0022-1759(00)00238-6

[34] Dupont NC, Wang K, Wadhwa PD, Culhane JF, Nelson EL. Validation and comparison of luminex multiplex cytokine analysis kits with ELISA: determinations of a panel of nine cytokines in clinical sample culture supernatants. J Reprod Immunol. 2005;66: 175–191. 16029895 10.1016/j.jri.2005.03.005 PMC5738327

[35] Elshal MF, McCoy JP. Multiplex bead array assays: performance evaluation and comparison of sensitivity to ELISA. Methods. 2006;38: 317–323. 16481199 10.1016/j.ymeth.2005.11.010 PMC1534009

[36] World Health Organization. Obesity: preventing and managing the global epidemic. Report of a WHO consultation. World Health Organ Tech Rep Ser. 2000;894: i–253. 11234459

[37] Hollingshead AB. Four factor index of social status New Haven, USA: Yale University Press; 1975.

[38] Dufour MC. What is moderate drinking? Defining drinks and drinking levels. Alcohol Res Health. 1999;23: 5–14. 10890793 PMC6761695

[39] Giovannucci E, Colditz G, Stampfer MJ, Rimm EB, Litin L, Sampson L, et al The assessment of alcohol consumption by a simple self-administered questionnaire. Am J Epidemiol. 1991;133: 810–817. 2021148 10.1093/oxfordjournals.aje.a115960

[40] O'Donovan A, Neylan TC, Metzler T, Cohen BE. Lifetime exposure to traumatic psychological stress is associated with elevated inflammation in the Heart and Soul Study. Brain Behav Immun. 2012;26: 642–649. 10.1016/j.bbi.2012.02.003 Epub 2012 Feb 15. 22366689 PMC3322304

[41] Perneger TV. What’s wrong with Bonferroni adjustments. BMJ. 1998;316: 1236–1238. 9553006 10.1136/bmj.316.7139.1236 PMC1112991

[42] Rothman KJ. No adjustments are needed for multiple comparisons. Epidemiology. 1990;1: 232–238. 2081237

[43] Bekker MH, van Mens-Verhulst J. Anxiety disorders: sex differences in prevalence, degree, and background, but gender-neutral treatment. Gend Med. 2007;4 Suppl B: S178–193. 18156102 10.1016/s1550-8579(07)80057-x

[44] Roest AM, Martens EJ, de Jonge P, Denollet J. Anxiety and risk of incident coronary heart disease: a meta-analysis. J Am Coll Cardiol. 2010;56: 38–46. 10.1016/j.jacc.2010.03.034 20620715

[45] Emerging Risk Factors Collaboration, Kaptoge S, Di Angelantonio E, Lowe G, Pepys MB, Thompson SG, et al C-reactive protein concentration and risk of coronary heart disease, stroke, and mortality: an individual participant meta-analysis. Lancet. 2010;375: 132–140. 10.1016/S0140-6736(09)61717-7 20031199 PMC3162187

[46] Kaptoge S, Seshasai SR, Gao P, Freitag DF, Butterworth AS, Borglykke A, et al Inflammatory cytokines and risk of coronary heart disease: new prospective study and updated meta-analysis. Eur Heart J. 2014;35: 578–589. 10.1093/eurheartj/eht367 Epub 2013 Sep 10. 24026779 PMC3938862

[47] Libby P, Simon DI. Inflammation and thrombosis: the clot thickens. Circulation. 2001;103: 1718–1720. 11282900 10.1161/01.cir.103.13.1718

[48] Libby P. Inflammation in atherosclerosis. Nature. 2002;420: 868–874. 12490960 10.1038/nature01323

[49] Steptoe A, Kivimäki M. Stress and cardiovascular disease. Nat Rev Cardiol. 2012; 9: 360–370. 10.1038/nrcardio.2012.45 22473079

[50] von Känel R. Psychosocial stress and cardiovascular risk: current opinion. Swiss Med Wkly. 2012;142: w13502 10.4414/smw.2012.13502 22271452

[51] Dantzer R, Kelley KW. Twenty years of research on cytokine-induced sickness behavior. Brain Behav Immun. 2007;21: 153–160. 17088043 10.1016/j.bbi.2006.09.006 PMC1850954

